# Using a partial atomic model from medium-resolution cryo-EM to solve a large crystal structure

**DOI:** 10.1107/S2059798320015156

**Published:** 2021-01-01

**Authors:** Montserrat Fàbrega-Ferrer, Ana Cuervo, Francisco J. Fernández, Cristina Machón, Rosa Pérez-Luque, Joan Pous, M. Cristina Vega, José L. Carrascosa, Miquel Coll

**Affiliations:** a Institute for Research in Biomedicine (IRB Barcelona), The Barcelona Institute of Science and Technology, Baldiri Reixac 10, 08028 Barcelona, Spain; b Institut de Biologia Molecular de Barcelona (IBMB–CSIC), Baldiri Reixac 10, 08028 Barcelona, Spain; c Centro Nacional de Biotecnología (CNB–CSIC), Darwin 3, 28049 Madrid, Spain; d Centro de Investigaciones Biológicas (CIB–CSIC), Ramiro de Maeztu 9, 28040 Madrid, Spain

**Keywords:** molecular replacement, cryo-EM, density modification, bacteriophage portal

## Abstract

Medium-resolution cryo-electron microscopy maps, in particular when they include a significant number of α-helices, may allow the building of partial models that are useful for molecular-replacement searches in large crystallo­graphic structures when the structures of homologs are not available and experimental phasing has failed. Here, as an example, the solution of the structure of a bacteriophage portal using a partial 30% model built into a 7.8 Å resolution cryo-EM map is shown. Inspection of the self-rotation function allowed the correct oligomerization state to be determined, and density-modification procedures using rotation matrices and a mask based on the cryo-EM structure were critical for solving the structure. A workflow is described that may be applicable to similar cases and this strategy is compared with direct use of the cryo-EM map for molecular replacement.

## Abstract 2

A partial 30% atomic model from a 7.8 Å resolution cryo-EM volume was used to phase X-ray data by molecular replacement.

## 1. Introduction   

X-ray crystallography is the technique that has provided the most high-resolution information in the field of structural biology. Although nowadays it is considered to be a well established technique, solving the structures of certain samples, such as large complexes, continues to be a challenge. Experimental phasing strategies by isomorphous replacement and anomalous diffraction may be time-consuming or may fail when well diffracting crystals or their derivatives are difficult to obtain. In many cases, molecular replacement (MR) becomes the best, or even the only, option for solving these types of structures. This method requires the availability of a structurally similar model (Rossmann & Blow, 1962 ▸).

In recent years, single-particle cryo-electron microscopy (cryo-EM) has experienced a resolution revolution (see Nogales, 2016 ▸, and references cited therein). The development of direct electron detectors and the availability of new processing programs, such as *MotionCor*2 (Zheng *et al.*, 2017 ▸) and *RELION* (Scheres, 2016 ▸), have paved the way to obtaining atomic models which can be directly built into the high-resolution cryo-EM maps. Indeed, cryo-EM has some methodological advantages when compared with X-ray crystallography: it requires lower amounts of sample, it avoids the crystallization bottleneck and it is able to deal with heterogeneous samples. However, the process of obtaining a high-resolution cryo-EM structure may still be quite arduous, including steps that are hard to automate, such as grid preparation and data processing (Doerr, 2016 ▸). In many cases, achieving map resolutions that allow the full tracing of atomic models is not straightforward. Consequently, challenging projects remain stuck in the intermediate-resolution range. In difficult cases where one technique alone is not able to succeed, combining data from cryo-EM and X-ray crystallo­graphy may be an effective strategy.

The structural study of viral capsids with icosahedral symmetry, in which intermediate-resolution cryo-EM reconstructions were used as phasing models, has contributed significant methodological advances (Stuart & Abrescia, 2013 ▸). This approach takes advantage of the icosahedral symmetry present in the samples and uses symmetrized maps. After the cryo-EM resolution revolution, similar procedures applicable to samples without such high orders of symmetry have also been developed (Xiong, 2008 ▸; Jackson *et al.*, 2015 ▸; Zeng *et al.*, 2018 ▸). In these approaches, the cryo-EM map was used directly as an initial model for density modification.

In this article, we show a case example in which a combination of the X-ray crystallography and cryo-EM techniques can be used in a different way, using a partial cryo-EM atomic model instead of the cryo-EM map for MR. Both strategies appear to be equally valid in our example, with that described here being a possible alternative in the case of the failure of direct use of the cryo-EM map.

The bacteriophage portal protein (also named connector) is found at a unique vertex of the viral capsid and is essential for procapsid assembly, genome encapsidation, tail assembly and genome ejection. Its overall architecture corresponds to a ring-like hollow cylindrical dodecamer (Cuervo & Carrascosa, 2012 ▸). However, portals have also been described to be able to assemble as undecameric or tridecameric complexes after overexpression, although they are only incorporated into the procapsids as dodecamers. This heterogeneity implies an additional difficulty in their structural characterization (Sun *et al.*, 2015 ▸). The portal protein of the T7 bacteriophage is coded by the *gp8* gene and has a predicted molecular weight of 59 kDa, which would give a multimeric complex of 650–770 kDa, depending on its oligomerization state.

## 2. X-ray crystallographic preliminary studies   

### 2.1. Crystallization   

The gp8 protein was expressed, purified and crystallized as described previously (Cuervo *et al.*, 2019 ▸). Data set 1, presented here and which yielded PDB entry 6tjp, was obtained from a bar-shaped crystal grown by the hanging-drop vapour-diffusion technique at 293 K using a 4.4 mg ml^−1^ protein sample in the following conditions: 0.2 *M* CaCl_2_, 0.1 *M* HEPES pH 7.5, 18%(*w*/*v*) PEG 400. The crystal was cooled in the same condition with 30%(*w*/*v*) PEG 400 as a cryoprotectant and kept in liquid nitrogen until X-ray data collection. Data set 2 yielded the gp8_closed_ structure (PDB entry 6qx5), and details of its crystallization and data-collection statistics have previously been published (Cuervo *et al.*, 2019 ▸).

### 2.2. Data collection and analysis   

For data set 1, X-ray data were collected on beamline ID14-4 at the European Synchrotron Radiation Facility (ESRF), Grenoble, France. Diffraction data were indexed and integrated with *XDS* and scaled, reduced and merged using *XSCALE* (Kabsch, 2010 ▸). Although a total of 270 images were collected, the statistics improved significantly when considering only the first 125 images (Table 1 ▸). All of the following X-ray data analyses were carried out using the *CCP*4 suite of crystallographic programs (Winn *et al.*, 2011 ▸).

#### 2.2.1. Oligomerization state and self-rotation function   

**Figure 1:**
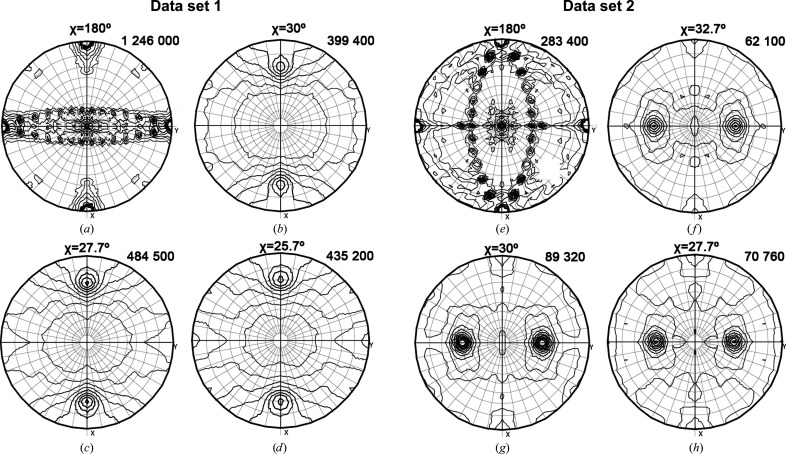
Sterographic projections of SRFs of two distinct *P*2_1_2_1_2_1_ data sets with maxima indicated. Projections of data set 1 show that the NCS is 13 (484 500) (*c*). In (*a*), the 1 246 000 peak corresponds to the crystallographic twofold axis; further peaks close to the equator correspond to 13 twofold axes perpendicular to the 13-fold axis shown in (*c*). Projections of data set 2, however, show that the NCS is 12 (89 320) (*g*). In (*e*), 12 peaks correspond to twofold axes perpendicular to the 12-fold axis shown in (*g*).

Matthews coefficient (*V*_M_) calculations on both data sets suggested one portal oligomer per asymmetric unit, but these calculations were not conclusive for determining the number of protomers of each portal oligomer. Nevertheless, self-rotation function (SRF) calculations performed with *MOLREP* (Vagin & Teplyakov, 2010 ▸) indicated that the T7 portal was composed of 13 protomers in the data set 1 crystal. Figs. 1(*a*), 1(*b*), 1(*c*) and 1(*d*) ▸ show different stereographic projections of the SRF at χ = 180°, χ = 30°, χ = 27.7° and χ = 25.7°. Comparing the peaks at χ = 30°, χ = 27.7° and χ = 25.7°, which would correspond to the presence of dodecameric, tridecameric or tetradecameric NCS, respectively, the highest peak was found to be in the χ = 27.7° section. Consistent with this observation, there were 13 peaks in the χ = 180° section perpendicular to the 13-fold axis. Therefore, the complex present in the crystal was a tri­decamer, with one ring per asymmetric unit and with a solvent content of 49%.

A similar analysis performed with data set 2 showed that it corresponded to a dodecameric form of gp8 (Figs. 1 ▸ *e*, 1 ▸ *f*, 1 ▸ *g* and 1 ▸ *h*). In this case comparison of the SRF peaks at χ = 32.7°, χ = 30° and χ = 27.7° revealed the highest peak at section χ = 30°, which corresponds to a 12-fold NCS axis. Moreover, there were 12 peaks at χ = 180°, which were perpendicular to the 12-fold axis. Thus, data set 2 consisted of a single dodecameric ring per asymmetric unit, with a solvent content of 57%.

## 3. Structure solution   

Experimental phasing was attempted extensively both with selenomethionine-derivative protein crystals and heavy-atom or cluster soaking. However, well diffracting derivative crystals could not be obtained. Moreover, no model with sufficient similarity to the gp8 protein to perform MR was available. Therefore, a new strategy was planned, which consisted of structural characterization of the sample by cryo-EM.

### 3.1. Using a medium-resolution cryo-EM map to obtain an initial model   

**Figure 2:**
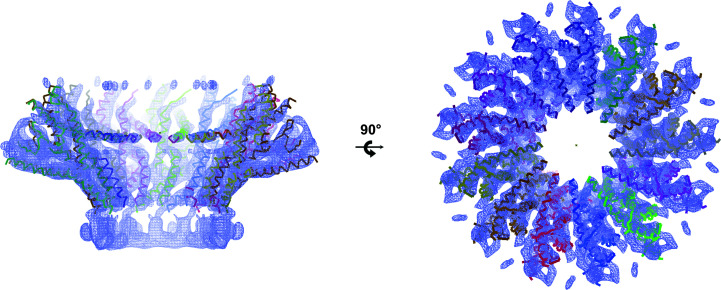
Tridecameric partial α-helical polyalanine model built on the 7.8 Å resolution cryo-EM map.

Single-particle cryo-EM data were initially collected using a Talos Arctica microscope (Cuervo *et al.*, 2019 ▸; Table 2 ▸). Data processing was challenging owing to the heterogeneity of the sample, which contained a mixture of different oligomeric states, and the lack of lateral orientations. Eventually, the 3D classification of a subset of 1200 particles with *RELION* (Scheres, 2016 ▸) yielded a map of a tridecameric portal at 7.8 Å resolution. *Coot* (Emsley *et al.*, 2010 ▸) was then used to interpret the map and to build a preliminary partial gp8 monomeric model. Built as polyalanine chains, the model consisted of nine α-helices. It contained 194 residues of the total of 536 amino acids present in the gp8 monomer. The sequence of the residues could not be established because the connectivity between the α-helices was not clear and their direction was difficult to determine. Once the partial monomeric model had been built, a tridecameric partial model was constructed, applying rotation matrices and a translation vector to account for the 13-fold axis running along the centre of the particle channel. We used standard rotation matrices with an angle of 2π/13 around the model axis and the corresponding translation to keep the model centred on the EM volume. This calculation was implemented in a short gawk script that also updated the chain names. The 13-fold ring model was then real-space refined against the 7.8 Å resolution cryo-EM map with *Phenix* (Fig. 2 ▸; Afonine *et al.*, 2018 ▸).

### 3.2. Molecular replacement   

**Figure 3:**
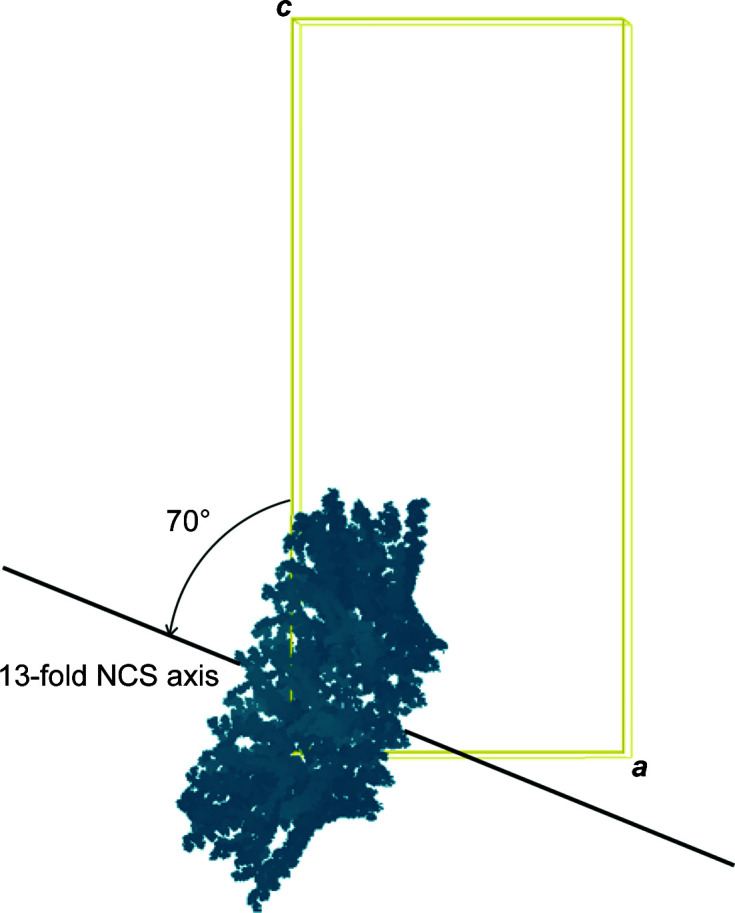
Localization of the MR solution in the *P*2_1_2_1_2_1_ crystal unit cell visualized with *Coot*. The unit cell is represented as a rectangle. The 13-fold NCS axis is located on the *ac* plane inclined 70° with respect to *c*.

The resulting tridecameric partial model was used for MR with *Phaser* (McCoy *et al.*, 2007 ▸) against data set 1. A unique solution was found with a positive log-likelihood gain (LLG) of 96 and a final translation-function *Z*-score value of 12.2, which indicated that the structure had been solved. The orientation of the symmetry axes of order 13 agreed with the outcome of the SRF. The peak in the χ = 27.7° section appeared at θ = 70°, φ = 0°, which indicated that the NCS axis of order 13 was located on the *XZ* (or *ac*) plane, inclined 70° from the *Z* (*c*) axis (Fig. 3 ▸). The model was then subjected to rigid-body refinement by protomer using *REFMAC*5 (Murshudov *et al.*, 2011 ▸), which moved all of the protomers significantly (3.5 Å) towards the central channel, shrinking the particle diameter and central channel. This observation led us to suspect that the pixel size given for the cryo-EM data was not accurate, as was confirmed later by the microscope facility. We initially were given a pixel size of 1.42 Å per pixel, while a later calibration of the instrument gave 1.37 Å per pixel. The magnification-factor error could be a serious issue when using cryo-EM data for MR. In our case, such an error translated into a shift of more than 6.5 Å in the diameter of an object of approximately 180 Å. However, phasing with the partial model was still successful, probably because the built helices were mostly not located at the edge of the particle.

### 3.3. Density modification and model building   

**Figure 4:**
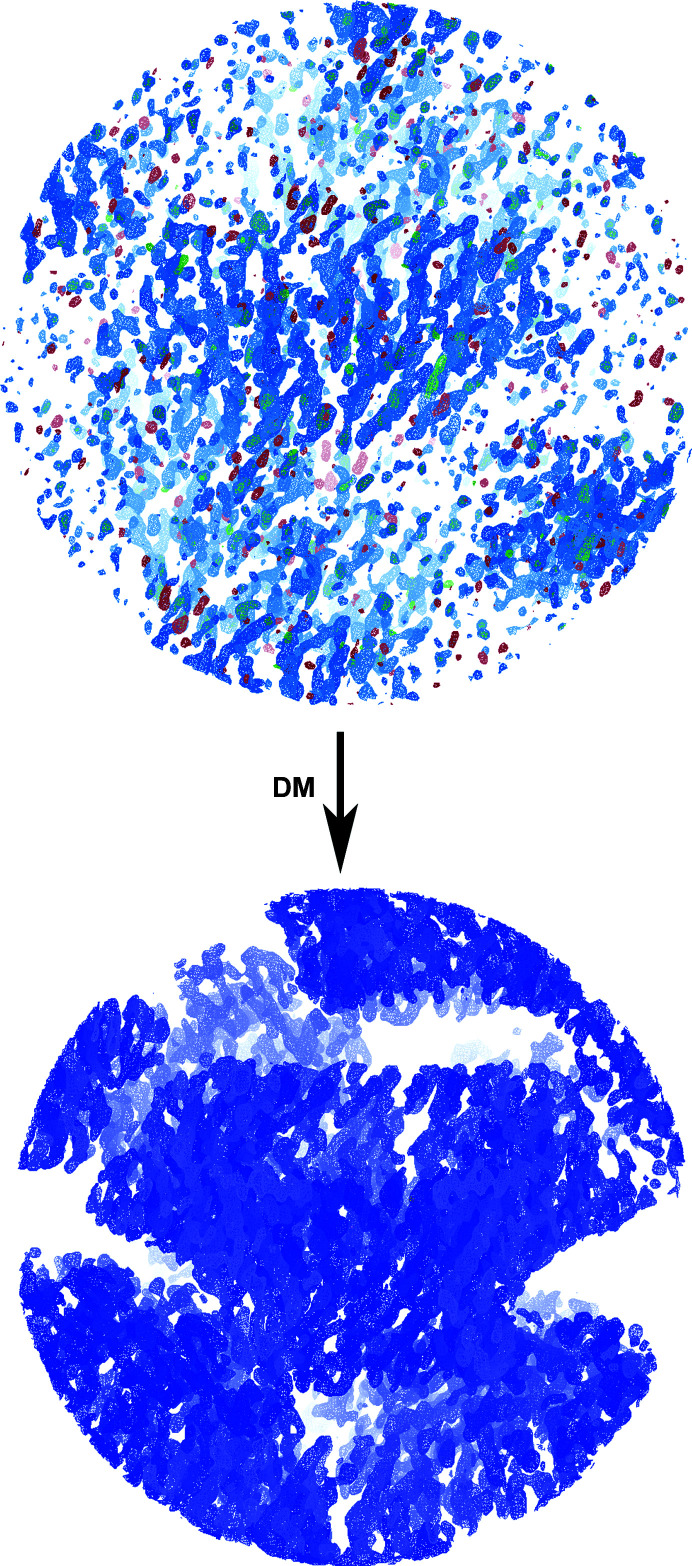
Improvement of the MR map by DM and phase-extension procedures. Top image: electron-density map after MR; the 2*F*_o_ − *F*_c_ map is represented in blue and the *F*_o_ − *F*_c_ map is depicted in green (positive) and red (negative). Bottom image: map after DM.

**Figure 5:**
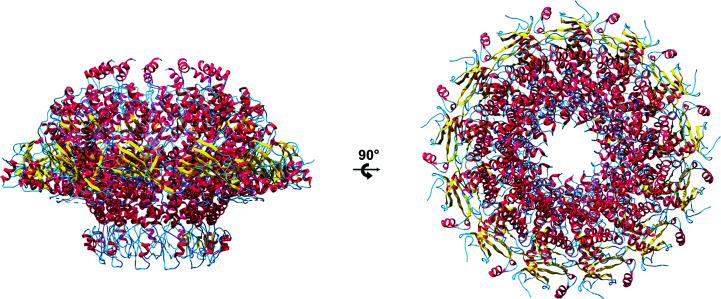
Side (left) and top (right) views of the gp8 tridecamer. The protein structure is depicted in cartoon representation with α-helices in red, β-strands in yellow and coils in blue.

After rigid-body refinement, the MR map showed that the partial α-helical model fitted well into the electron density, but features further away from the initial model were not interpretable (Fig. 4 ▸, upper panel). Density-modification (DM) procedures were critical in order to improve the map (Cowtan, 2010 ▸). Solvent flattening, histogram matching and NCS averaging were applied with masks generated from the cryo-EM map. A mask of the whole complex was used for solvent flattening, while a slice of it comprising 27.7° of the portal map was used for NCS averaging. The same rotation matrices as used to build the cryo-EM atomic tridecamer were applied. A number of variations were made to the setting parameters and the best conditions were identified in terms of final average NCS correlation: a phase-extension protocol by resolution steps, starting at 7.9 Å, with solvent and averaging masks updated every 50 and 20 cycles, respectively, and a total of 104 cycles. The correlation between NCS-related regions of the map was 0.849. This DM procedure yielded a fully interpretable electron-density map of the particle (Fig. 4 ▸, lower panel). A model of the tridecameric protein could be built and refined. During model building, NCS-averaged maps calculated with *Coot* proved to be critical for the correct interpretation of the maps. Refinement of the structure yielded a tridecameric model containing 481 amino acids per monomer (Fig. 5 ▸, Table 3 ▸; PDB entry 6tjp).

## 4. Comparison with the direct use of a cryo-EM map for MR   

The direct use of medium-resolution cryo-EM maps (such as that described in Agirrezabala *et al.*, 2005 ▸) for phasing crystallographic data had previously been attempted without success. However, after solving the structure using the partial model, MR was again tried as an exercise with the new ‘post-resolution-revolution’ cryo-EM map, both with the inaccurate pixel size used during model building and with the corrected pixel size. In both cases a correct MR solution was obtained, with TFZ and LLG values of 16.0 and 81, respectively, in the first case and 26.7 and 546, respectively, in the second case. These values are better than when a partial model is used, in particular when the scale-factor error is corrected, but it has to be noted that only less than a third of the structure is used in the partial atomic model case.

**Figure 6:**
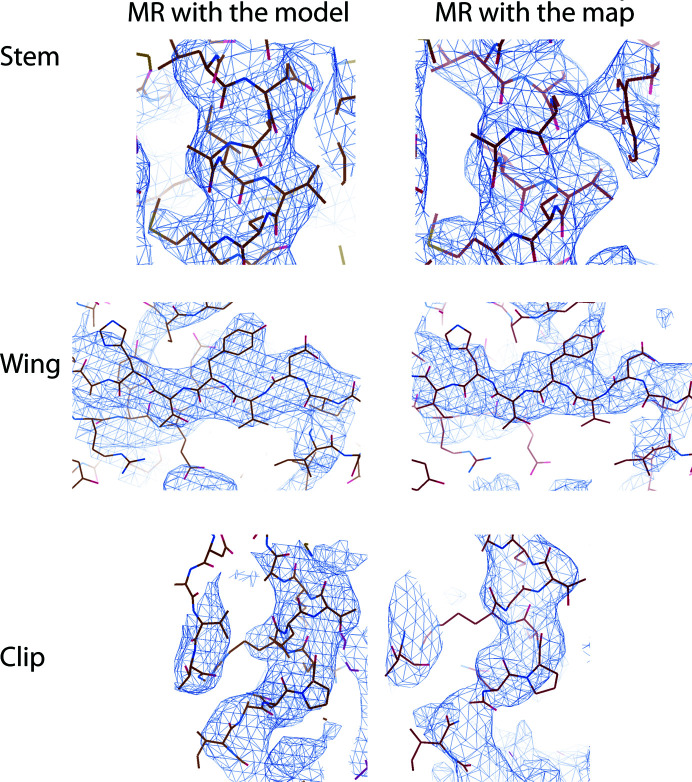
Comparison of the crystallographic 2*F*_o_ − *F*_c_ maps of three different protein domains after MR procedures either with the partial model or the cryo-EM map. Both options yield interpretable maps that allow model building. Images were obtained with *Coot*.

In addition, the final maps obtained by phasing with the partial model and with the cryo-EM map were also compared, showing that both of them have a similar level of detail in all of the domains, which would allow the building of the final model (Fig. 6 ▸). Thus, both strategies appear to give, in our example, the correct solution.

## 5. Solution of the physiological dodecameric gp8 atomic model   

The monomeric structure of gp8 was used to solve the structure of the protein in its physiological dodecameric form using data set 2 and placing 12 copies of the monomer by MR (Cuervo *et al.*, 2019 ▸). All structures were refined with* REFMAC*5 and *Phenix* (Liebschner *et al.*, 2019 ▸), first applying tight NCS restraints, which were progressively relaxed on the side chains. All models were validated with *MolProbity* (Williams *et al.*, 2018 ▸).

## 6. Discussion   

Cryo-EM maps have successfully been used to directly phase X-ray data by MR (Wynne *et al.*, 1999 ▸; Chandran *et al.*, 2009 ▸; Song *et al.*, 2015 ▸). Here, we describe an alternative protocol that also combines X-ray crystallography and cryo-EM data for solving macromolecular structures. Information from the cryo-EM experiment is incorporated into the workflow in two specific steps: MR and DM.

The workflow we present here is based on building a partial model *de novo*, and therefore no high-resolution information such as a previous atomic model is required. When compared with the direct use of the cryo-EM map for MR, this strategy avoids the map-preparation steps required in these protocols (Jackson *et al.*, 2015 ▸). The extra step of building a partial model in *Coot* was fast using the automatic helix-building option in *Coot*, and thus does not increase the time effort.

**Figure 7:**
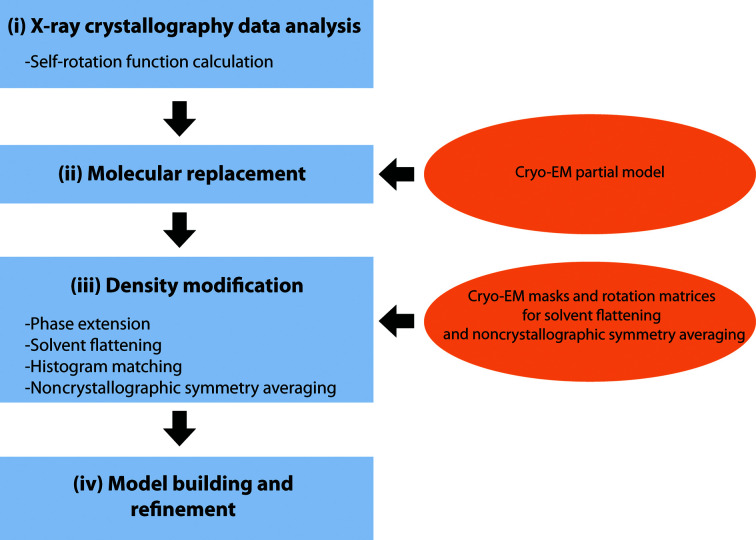
Protocol workflow used to solve the structure. Classical X-ray crystallography steps are depicted vertically in the blue squares on the left. Orange ellipses show the points where cryo-EM-derived information is incorporated into the protocol.

We suggest the following protocol as an alternative to the direct use of the cryo-EM map to phase crystallographic structures (Fig. 7 ▸).

(i) Carefully analyse the X-ray crystallographic data. The data should provide useful information about the composition of the asymmetric unit and the presence and orientation of NCS axes. This information is used in the following steps.
(ii) Perform MR with a partial atomic model of the protein built from a medium-resolution cryo-EM map. Well defined secondary-structure features such as α-helices are usually possible to build.
(iii) Perform DM protocols in order to improve the MR map and render interpretable regions away from the partial model. Use the cryo-EM map to obtain the rotation matrices and the masks for solvent flattening and NCS averaging.
(iv) Test different DM phase-extension protocols and choose the one that gives the highest correlation between NCS-related regions and the best interpretable and feature-completed map. Proceed to model building and refinement.

It is important to note that for DM calculation procedures the accurate orientation and location of the NCS axis, as well as an accurate mask from the cryo-EM volume, are necessary to allow the rapid and significant improvement of the crystallo­graphic electron-density map.

Using this protocol, we managed to phase X-ray data with a partial model of the protein containing only 30% of the residues built as polyalanine chains. Although we also managed to subsequently solve the structure by performing MR with the cryo-EM map as an initial model, we present this workflow as an alternative, which in our case yielded the correct solution in a short time.

## 7. Conclusions   

Despite its spectacular advances, cryo-EM may not always provide maps of sufficient resolution to allow the building and refinement of a full atomic model, depending on the behaviour of the sample. However, medium-resolution cryo-EM maps can be obtained rapidly and usually show clear secondary-structure features, in particular α-helices. On the other hand, large X-ray crystallographic structures can sometimes be difficult to determine because of a lack of derivatives or the long experimental procedures that are required before well diffracting crystals that are useful for phasing are obtained. As structural biology projects become more challenging, dealing with heterogeneous and large complexes, combining data from cryo-EM and X-ray crystallography emerges as an advantageous strategy. This can be performed by directly using the cryo-EM map as an initial model for MR or, as an alternative, by using a partial model built on the cryo-EM map as shown here.

## Figures and Tables

**Table 1:** Data-collection and processing statistics for data set 1 (PDB entry 6tjp) Values in parentheses are for the outermost shell.

Diffraction source	ID14-4, ESRF
Wavelength (Å)	0.9791
Temperature (K)	100
Detector	ADSC Q4r CCD
Crystal-to-detector distance (mm)	287.00
Rotation range per image (°)	0.75
Total rotation range (°)	75
Exposure time per image (s)	10
Space group	*P*2_1_2_1_2_1_
*a*, *b*, *c* (Å)	119.85, 238.57, 265.61
α, β, γ (°)	90, 90, 90
Mosaicity (°)	0.282
Resolution range (Å)	49.47–3.74 (3.87–3.74)
Total No. of reflections	303750 (28381)
No. of unique reflections	78414 (7216)
Completeness (%)	98.6 (91.7)
Multiplicity	3.9 (3.9)
〈*I*/σ(*I*)〉	9.1 (1.4)
*R*_meas_	0.188 (1.401)
CC_1/2_	0.993 (0.558)
Overall *B* factor from Wilson plot (Å^2^)	130.85

**Table 2:** Cryo-EM data collection

Microscope	Talos Arctica (FEI)
Voltage (kV)	200
Detector	Falcon II (FEI)
Nominal magnification	73000
Electron exposure (e^−^ Å^−2^)	22.8
Defocus range (µm)	−1.0 to −3.0
Pixel size (Å^2^ per pixel)	1.37
Symmetry imposed	*C*13
Initial No. of particle images	180911
Final No. of particle images	1200
Map resolution (Å)	7.8

**Table 3:** Refinement statistics for data set 1 (PDB entry 6tjp) Values in parentheses are for the outermost shell.

Resolution range (Å)	49.47–3.74
Completeness (%)	98.6
No. of reflections, working set	74388
No. of reflections, test set	3915
Final *R*_cryst_	0.244
Final *R*_free_	0.286
Cruickshank DPI	0.842
No. of non-H atoms	
Protein	48126
Ion	0
Ligand	0
Water	0
Total	48126
R.m.s. deviations	
Bonds (Å)	0.003
Angles (°)	0.69
Average *B* factors (Å^2^)	
Protein	151.90
Ion	—
Ligand	—
Water	—
Ramachandran plot	
Most favoured (%)	89.83
Allowed (%)	9.73
